# Associations between Viral Infection History Symptoms, Granulocyte Reactive Oxygen Species Activity, and Active Rheumatoid Arthritis Disease in Untreated Women at Onset: Results from a Longitudinal Cohort Study of Tatarstan Women

**DOI:** 10.3389/fimmu.2017.01725

**Published:** 2017-12-05

**Authors:** Marina I. Arleevskaya, Albina Z. Shafigullina, Yulia V. Filina, Julie Lemerle, Yves Renaudineau

**Affiliations:** ^1^State Medical Academy, Kazan, Russia; ^2^Kazan Federal University, Kazan, Russia; ^3^Laboratory of Immunology and Immunotherapy, INSERM U1227, Hôpital Morvan, Centre Hospitalier Régional Universitaire (CHU) de Brest, Brest, France

**Keywords:** rheumatoid arthritis, infection symptoms, viruses, women, reactive oxygen species

## Abstract

To evaluate the effects of infectious episodes at early stages of rheumatoid arthritis (eRA) development, 59 untreated eRA patients, 77 first-degree relatives, from a longitudinal Tatarstan women cohort, were included, and compared to 67 healthy women without rheumatoid arthritis (RA) in their family history. At inclusion, informations were collected regarding both the type and incidence of infectious symptom episodes in the preceding year, and granulocyte reactive oxygen species (ROS) were studied at the basal level and after stimulation with serum-treated zymosan (STZ). In the eRA group, clinical [disease activity score (DAS28), health assessment questionnaire] and biological parameters associated with inflammation (erythrocyte sedimentation rate, C-reactive protein) or with RA [rheumatoid factor, anticyclic citrullinated peptide (anti-CCP2) antibodies] were evaluated. An elevated incidence of infection events in the previous year characterized the eRA and relative groups. In addition, a history of herpes simplex virus (HSV) episodes was associated with disease activity, while an elevated incidence of anti-CCP2 autoantibody characterized eRA patients with a history of viral upper respiratory tract infection symptoms (V-URI). Granulocyte ROS activity in eRA patients was quantitatively [STZ peak and its area under the curve (AUC)] and qualitatively (STZ time of peak) altered, positively correlated with disease activity, and parameters were associated with viral symptoms including HSV exacerbation/recurrence, and V-URI. In conclusion, our study provides arguments to consider a history of increased viral infection symptoms in RA at the early stage and such involvement needs to be studied further.

## Introduction

Rheumatoid arthritis (RA) development results from an inappropriate immune response to environmental challenges in genetically predisposed patients. Accordingly, the list of microorganisms associated with RA is still growing, and several hypotheses support their causative role, as recently reviewed (1). On one hand, the immunological hypothesis proposes that RA development results from an inappropriate immune response to infections, which can lead to loss of tolerance to self-antigens. In support of such a hypothesis, up to 40 risk factors have been associated with RA, and a significant part of the genetic contribution is associated with the major histocompatibility complex locus (2). While on the other hand, the environmental hypothesis proposes that RA development may result from a cumulative effect of microbial/viral/environmental factors and thus explains the absence of a single defined pathogen. In this case, environmental factors may be accelerators or protective (3).

In order to decipher the respective contributions of the immune system and infections to RA, the longitudinal Tatarstan women cohort study was established in 2002 in order to include the follow-up of RA families (4). Our preliminary results from this cohort have established, first, an elevated rate of infection symptoms in the year preceding RA onset. Second, there is an elevated rate of bacterial colonization in feces, urine, and skin that remains after several years in treated patients with RA. Third, there are impaired innate (phagocyte) and acquired (antibacterial IgG response) immune responses in RA patients. In this study, our main objective was to study the infectious spectrum preceding RA onset and to test its impact on disease activity and innate immunity in comparison to controls and healthy first-degree relatives.

## Materials and Methods

### Subjects

In this prospective study conducted between 2002 and 2016 at the Kazan Rheumatology department, RA women were included in the Tatarstan cohort at time zero when they met RA diagnostic criteria according to the 2010 ACR/EULAR classification criteria (5, 6). In the cases of 14 of the patients who were diagnosed before 2010, the RA onset was diagnosed by consensus of three experienced rheumatologists. For this study, a total of 208 women were studied semiannually including 59 therapy naïve early stage patients [early stages of rheumatoid arthritis (eRA), defined as <6 months of RA duration, time since diagnosis 0.3 ± 0.1 years; age range: 40.8 ± 16.2 years old], 77 first-degree relatives, and a healthy control group (control) comprised of 67 women with no chronic disease and no RA among close relatives. All the individuals included in this study were described previously (4). Exclusion criteria were based on risk factors for infection as previously discussed (7). The study was approved by the Ethical Committee of the Kazan State Medical Academy, Kazan, Russia (Permit nr 1/2002). Consent was received from all patients involved in the study, including consent to participate in the study and consent to allow publication of the results.

When the RA diagnosis was verified and before starting therapy on the day of the chemiluminescent study, we also performed a standard clinical and laboratory examination including the erythrocyte sedimentation rate (ESR)-based 28 joints disease activity score (DAS28), the Health Assessment Questionnaire (HAQ), C-reactive protein (CRP), rheumatoid factor (RF), and antibodies to citrullinated peptides (CCP2), as previously described (8–10). All patients and those relatives with joint symptoms (pain and morning stiffness) in the small joints of the feet and hands underwent magnetic resonance imaging. Patients with a DAS28 ≥5.1 at the baseline were defined as highly active (11). The control group showed no symptoms of arthritis, no ESR, or CRP elevation (except during infection), and no reactivity of RF and anticyclic citrullinated peptide (anti-CCP2).

### Infection

Information on infections within the last year and according to the criteria defined in Table 1 was collected from the RA patients during 2-day hospital visits by a physician qualified in rheumatology (AMI). The subjects were biannually or annually questioned about any symptoms suggestive of infections experienced during the 6–12 months, and infection histories for the two semesters were compiled. For the control group, information was from the 1 year preceding enrollment. Information from documents at other clinics was sought whenever a general practitioner had been visited. All subjects were tested and were negative for serological markers of chronic viral infections including human viral hepatitis (HAV, HBV, and HCV), and human immunodeficiency virus (HIV). A history of allergic disease and/or detection of allergen-specific IgE was considered as an exclusion criterion.

**Table 1:** Criteria used in the study to establish infectious episodes within the last year.

Infection	Information about the diagnosis
V-URI or viral suspected upper respiratory tract infection (international classification of diseases code J06)	The following typical infectious episode criteria, known to be mainly of viral origin were used: – Catarrhal phenomena – Infection gradually developing with a prodromal period in the form of increasing symptoms of malaise, low-grade fever, headache, myalgia, arthralgia – Infection lasting 3–14 days (in cases not complicated by secondary bacterial infections) – Infection developed as a result of contact with infected persons, as well as after general and local cooling, overheating, emotional/mental, and physical overload – Therapy carried out with antiviral, and optionally antipyretic drugs
	Exclusion criteria: – Sudden and rapid development after contact with known allergens or potential allergens or while receiving either of the drugs – Itchy skin rashes in the form of urticaria, angioedema simultaneously with catarrhal phenomena. – Therapy carried out with antihistamines
	Information obtained from outpatient medical history records on infectious episodes in cases when the person is referred to a general practitioner. In other cases information was collected from anamnesis data

B-URI or bacterial suspected upper respiratory tract infection requiring antibiotic therapy	The diagnosis was made by a general practitioner who treated the patient in uncomplicated cases. With a more severe/protracted process the diagnosis was confirmed by an ear, nose, and throat (ENT) doctor who treated the patient. In all cases, including those determined by us, the diagnosis was verified based on the clinical, laboratory and instrumental examination according to the current standards of diagnosis and treatment of the disease and the drug therapy was performed with antibiotics or local antiseptic agents (but not antihistamines)
	Source of information—outpatient medical history record and collected anamnesis data

Acute bronchitis	The diagnosis was made by a general practitioner who treated the patient in uncomplicated cases. With a more severe/protracted process, the diagnosis was verified by a pulmonologist who treated the patient. In all cases, the diagnosis was verified based on the clinical, laboratory, and instrumental examination according to the current standards of diagnosis and treatment of the disease, and drug therapy was performed with antibiotics (but not antihistamines, exclusion criteria)
	Source of information: outpatient medical history card and collected anamnesis data

Herpes simplex virus (HSV) exacerbation/reactivation	The frequency and duration of HSV exacerbation/reactivation was retrospectively evaluated based on the questioning regarding typical clinical manifestations: – Blisters mainly on the lip and nose mucosa after super cooling, lack of sleep, mental stress, the effect of local antiviral therapy (but not antihistamines, exclusion criteria)
	In the solitary cases of herpetic stomatitis or keratitis or atypical dermal or mucosal localization of the blisters, the diagnosis was verified by an ENT doctor, dentist, dermatologist, or oculist based on the clinical, laboratory, and instrumental examination according to the current standards of diagnosis and treatment of the disease – HSV genital infection exacerbations were verified by a gynecologist based on the clinical, laboratory and instrumental examination according to the current standards of diagnosis and treatment of the disease
	Source of information—collected anamnesis data and outpatient medical history record

Chronic tonsillitis exacerbation	The diagnosis was confirmed by an ENT doctor who examined and treated the patient according to the current standards of diagnosis and treatment of the disease. In all the cases, fixed by us, drug therapy was performed with antibiotics or local antiseptic agents (but not antihistamines, exclusion criteria)
	Source of information: outpatient medical history card

No infection within the last year	Declared by the individual no clinical manifestation of any infection within the last year
	Source of information: collected anamnesis data

The following parameters of the infectious syndrome were analyzed: no infection, viral upper respiratory tract infection symptoms (V-URI), bacterial upper respiratory tract infection symptoms requiring antibiotic therapy (B-URI), herpes simplex virus (HSV) exacerbation/reactivation, chronic tonsillitis exacerbation, and acute bronchitis. The incidence of infectious episodes and their overall duration per year were also collected. Only those episodes judged by the rheumatologist to truly indicate an infection were scored. In the case of exacerbation of a chronic infection and/or doubt regarding an infection, the diagnosis was established by a specialist in the corresponding medical area.

### Granulocyte Reactive Oxygen Species (ROS)

Peripheral blood granulocytes were collected from 59 untreated and eRA patients, 77 relatives, and 58 controls. Since an active infection may introduce a bias in the analysis, samples from all groups were collected in a period when there were no clinical symptoms of an infection and without any routine laboratory signs of inflammation. Granulocytes were isolated on a Ficoll–Urografin density gradient. The proportion of granulocytes in the cell suspension was 90–95% and the percentage of viable cells was 95–98%. The production of ROS was assessed at the basal level and after stimulation with serum-treated zymosan (STZ, Sigma) by the luminol-dependent chemiluminescence technique (chemiluminometer designed by Santalov BF, Pushchino, Russia). Real-time registration was performed every 4 s in thermostat plastic chambers with continuous mixing of granulocyte suspensions (sample volume 0.2 ml, cell density 10^6^ cells/ml, and concentration of STZ 0.25 mg/ml). The following parameters were measured: spontaneous level of ROS production [arbitrary units (au)], peak of ROS production after STZ (in au), area under the curve (AUC, in au × min), and time of occurrence of peak ROS production (min).

### Statistical Analysis

Continuous data are described as mean ± SEM. Differences among groups were analyzed by one-way ANOVA in a non-parametric test and the Dunn’s test was used for *post hoc* comparisons, or the Fisher’s exact test for categorical data. Following normality and equality of variance tests, nominal values were compared to controls using the Student’s *t*-test or alternatively by using a nonparametric test (Mann–Whitney rank sum test) with Bonferroni corrections. For correlation analysis, the Pearson’s coefficient *r* was calculated and Bonferroni corrections applied. *P*-values under 0.05 were considered significant. Statistical analyses were performed using GraphPad Prism 7.0 (La Jolla, CA, USA).

## Results

### Infections

**Figure 1:**
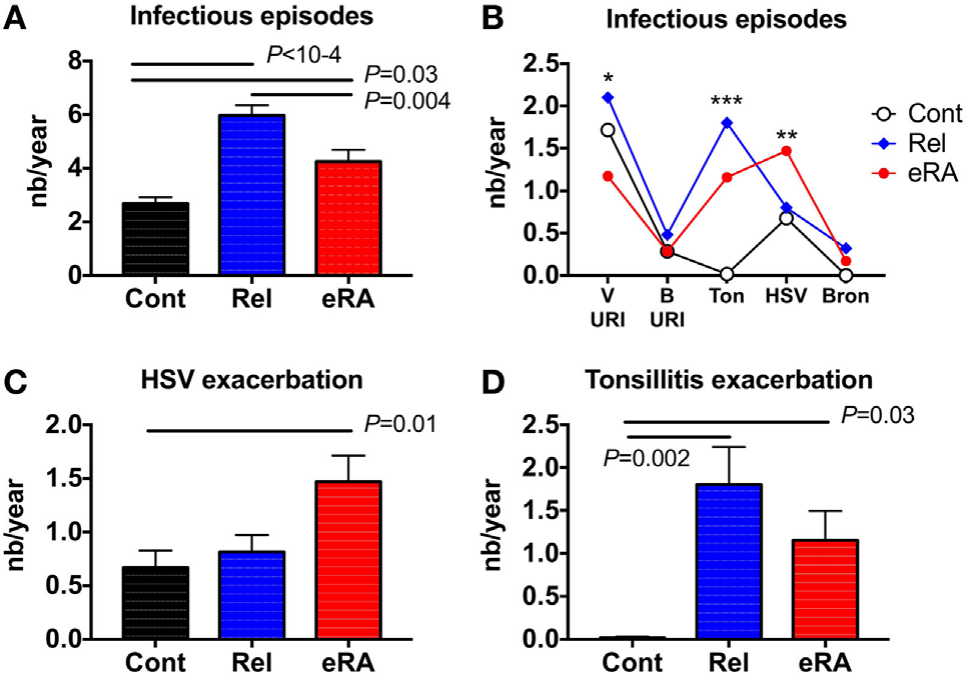
The number (nb) of infectious symptom events during the 1-year period preceding the arthritis onset (early [e]RA) or the first examination [healthy control (Cont) and first degree relatives (Rel)]. The following parameters were assessed: viral upper respiratory tract infection symptoms (V-URI), bacterial upper respiratory tract infection symptoms requiring antibiotic therapy (B-URI), acute bronchitis, herpes simplex virus (HSV) exacerbation/reactivation, and chronic tonsillitis exacerbation. **(A)** Total infectious symptom event incidence in the preceding year; **(B)** specific infectious symptom incidence history; **(C)** HSV exacerbation/reactivation; and **(D)** chronic tonsillitis exacerbation. Statistics are indicated when *P* ≤ 0.05 and (*) denotes the calculated *P-*values *P ≤ 0.05, **P ≤ 0.01, ***P ≤ 0.001.

In order to evaluate the incidence of infectious episodes in the year preceding RA onset, 59 untreated eRA women and their first-degree relatives (*n* = 77) were selected and compared to 67 healthy controls. In these 203 women, the following parameters were assessed: viral upper respiratory tract infection symptoms (V-URI), bacterial upper respiratory tract infection symptoms (B-URI), acute bronchitis, HSV exacerbation or reactivation, and chronic tonsillitis exacerbation. When compared to controls and first-degree relatives (Figure 1A), eRA patients included in this study had more frequent infection symptoms in the previous year than healthy controls (4.2 ± 0.4 events/year in eRA versus 2.7 ± 0.2 in controls, *P* = 0.03), but less than what was observed in relatives (6.0 ± 0.4 events/year, *P* = 0.004 versus eRA, and *P* < 10^−4^ versus controls).

Next, and as presented in Figure 1B, the incidences of each specific type of infection in the subjects’ histories were compared between the three groups, revealing differences for V-URI (Kruskal–Wallis test, *P* = 0.04), HSV exacerbation/reactivation (*P* = 0.01), and chronic tonsillitis reactivation (*P* = 0.001). An increase for HSV in the infection exacerbation/reactivation history was observed in eRA patients (Figure 1C, 1.5 ± 0.2 events/year in eRA versus 0.7 ± 0.2 in controls and versus 0.8 ± 0.2 in relatives, *P* = 0.005 and *P* = 0.02, respectively). Chronic tonsillitis reactivation in the history was almost absent from controls, present in eRA and the highest prevalence was observed in relatives (Figure 1D, *P* = 0.03 and *P* = 0.002, respectively). When considering the V-URI history, there was a trend for V-URI incidence reduction in eRA patients (data not shown). As the incidence of B-URI was similar between the three groups, and as the prevalence and incidence of acute bronchitis was modest in relatives and in eRA patients, both parameters were not considered further.

### Clinical Data

Next, and as presented in Table 2, the impacts of V-URI, HSV exacerbation/reactivation, and chronic tonsillitis reactivation in the history of episodes were analyzed with regards to the clinical and biological parameters of RA. First, eRA patients who presented V-URI symptoms in the previous year were characterized by an elevated prevalence of anti-CCP2 autoantibodies (*P* = 0.006). Second, for those early stage patients who suffered from HSV infection exacerbations during the year preceding the examination, they had more active RA disease (DAS28 > 5.1, *P* = 0.0005), and no difference when considering HAQ, ESR, CRP, RF, and anti-CCP2 autoantibodies. Third, no associations were related to a chronic tonsillitis exacerbation episode history. These results support the notion that V-URI and HSV symptoms in the history influence RA activity, but probably through distinct pathways.

**Table 2:** Characteristics of early (e)RA patients according their infection symptoms reported in the previous year: all studied infection symptoms (All), viral upper respiratory tract infection symptoms (V-URI), Herpes simplex virus (HSV) exacerbation or reactivation, and chronic tonsillitis (Ton) exacerbation.

	All	V-URI−	V-URI+	HSV−	HSV+	Ton−	Ton+
DAS28	5.2 ± 0.2	4.9 ± 0.5	5.4 ± 0.2	4.8 ± 0.3	5.7 ± 0.2	5.3 ± 0.2	5.0 ± 0.7
DAS28 >5.1	37/59 (63%)	8/16 (50%)	29/43 (67%)	11/28 (39%)	26/31 (84%)***	29/48 (60%)	8/11 (73%)
Health assessment questionnaire	2.9 ± 0.3	4.1 ± 0.7	2.5 ± 0.2	3.1 ± 0.5	2.8 ± 0.3	3.0 ± 0.3	2.6 ± 0.5
Erythrocyte sedimentation rate	38 ± 2	39 ± 4	37 ± 2	33 ± 3	41 ± 2	38 ± 2	34 ± 6
C-reactive protein (mg/L)	25 ± 3	33 ± 6	22 ± 3	20 ± 4	29 ± 4	24 ± 3	28 ± 9
Rheumatoid factor (UI/L)	53 ± 8	23 ± 3	65 ± 11	61 ± 16	46 ± 7	60 ± 10	24 ± 5
RF+ (>12 UI/L)	46/59 (78%)	10/16 (62%)	36/43 (84%)	22/28 (79%)	24/31 (77%)	39/48 (81%)	7/11 (64%)
CCP2 UI/L	495 ± 119	235 ± 128	598 ± 155	622 ± 171	298 ± 139	595 ± 155	244 ± 125
CCP2+ (>20 UI/L)	23/28 (82.1%)	3/8 (37.5%)	20/20 (100%)***	14/17 (82%)	9/11 (82%)	17/20 (85%)	6/8 (75%)

*Mean ± SEM*.

Statistics *0.01 < P < 0.05; **0.001 < P < 0.01; ***0.0001 < P < 0.001.

### Granulocyte ROS

**Figure 2:**
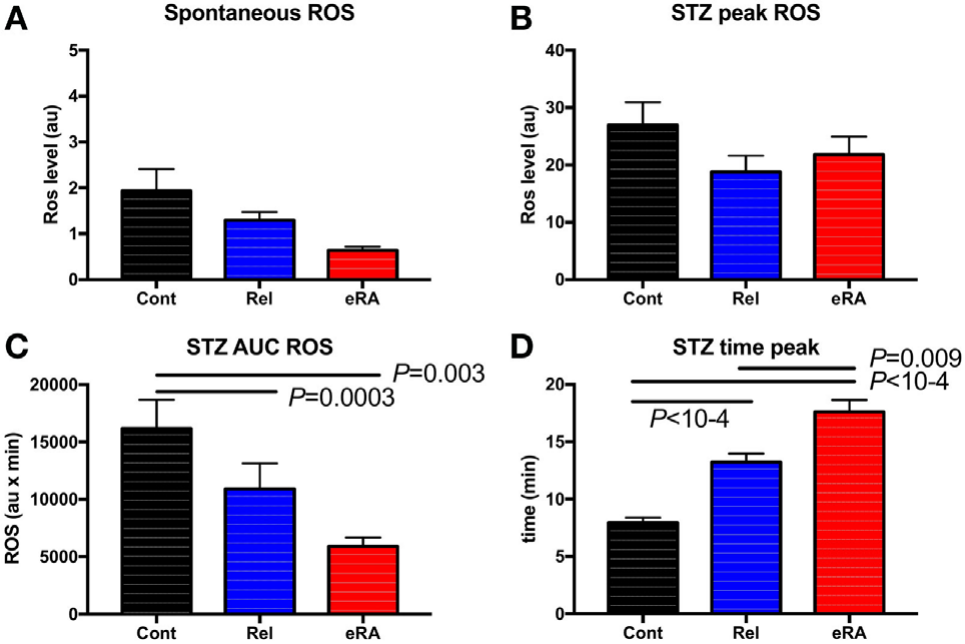
Granulocyte reactive oxygen species (ROS) production: spontaneous **(A)**, after serum treated zymozan (STZ) stimulation at peak **(B)**, area under the curve (AUC) **(C)**, and time to reach peak ROS production **(D)**. Healthy controls (Cont), rheumatoid arthritis (RA) first-degree relatives (Rel), and untreated RA patients at onset (early RA). Statistics are indicated when *P* ≤ 0.05.

Granulocyte ROS production plays a crucial role in controlling viral and bacterial infections and in the pathophysiology of RA by promoting inflammation and, in turn, by inducing cartilage and joint tissue damage. Accordingly, we measured the production of ROS at basal level (spontaneous), and after stimulation by using STZ in 59 eRA patients, 77 relatives, and 58 controls. In relatives and in eRA patients (Figures 2A,B), differences in relation to controls did not reach significance with regards to the basal level and the maximum peak of ROS production. In contrast (Figures 2C,D), for relatives and even more for eRA, a reduction in ROS AUC (5,924 ± 766 au × min in eRA, 10,927 ± 2,212 au × min in relatives versus 16,184 ± 2,489 au × min in controls, *P* = 0.003 and *P* = 0.0003, respectively) and a remarkably delayed time until the peak (17.6 ± 1.0 min in eRA versus 13.2 ± 0.7 min in relative and versus 7.9 ± 0.4 min in controls, both *P* < 10^−4^) were observed.

**Figure 3:**
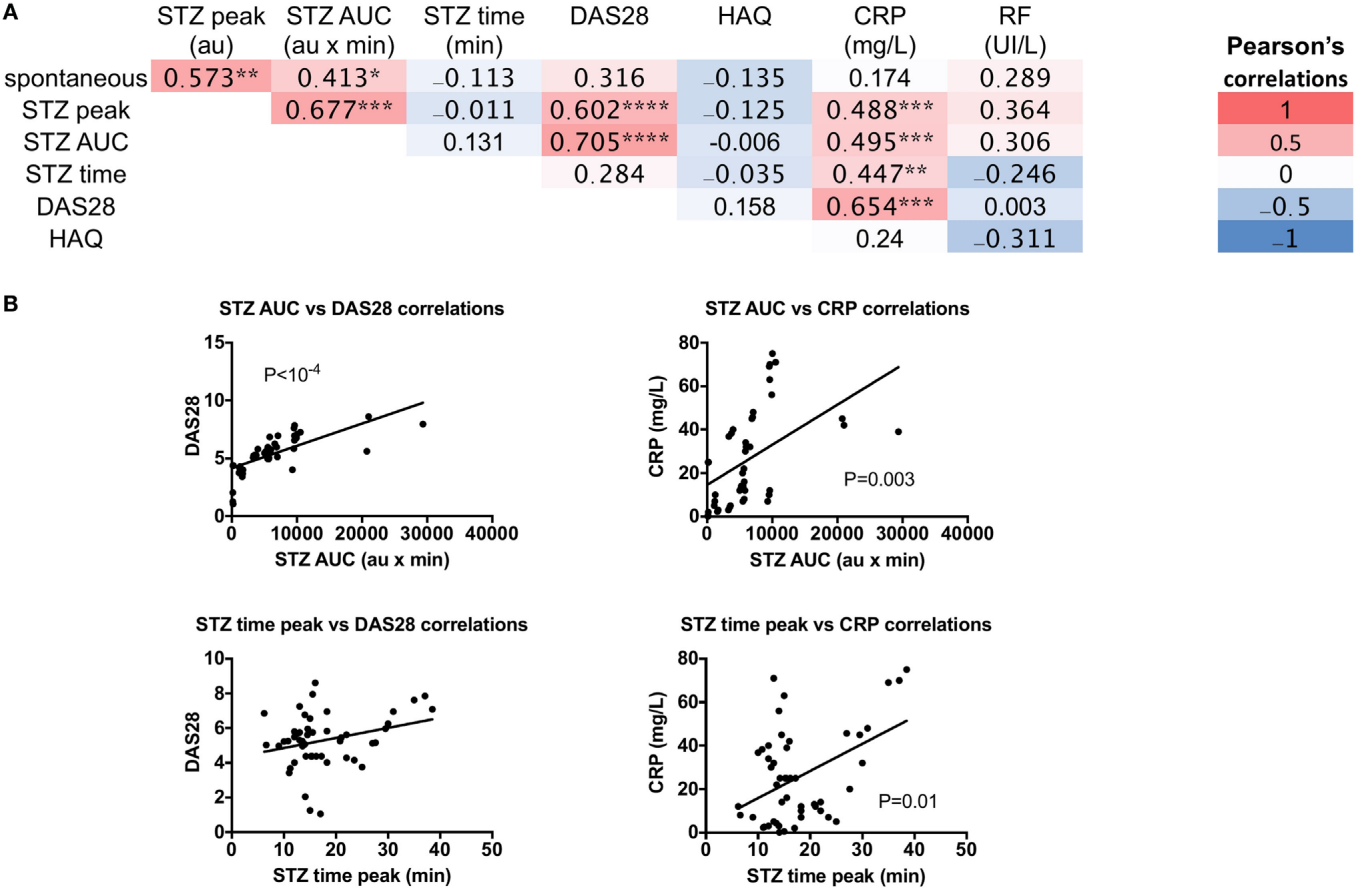
Correlations between granulocyte reactive oxygen species (ROS) production serum-treated zymozan (STZ) stimulation at peak, area under the curve (AUC), time to reach peak ROS production, and rheumatoid arthritis (RA) parameters: erythrocyte sedimentation rate-based 28 joints disease activity score (DAS28), the Health Assessment Questionnaire (HAQ), C-reactive protein (CRP), and rheumatoid factor (RF) levels in RA patients. **(A)** Correlation table of RA parameters. Colors represent the correlation coefficients (red being the highest and blue the lowest), whereas statistical significances (*) denotes the calculated *P* values *P ≤ 0.05, **P ≤ 0.01, ***P ≤ 0.001, and ****P ≤ 10^−4^. **(B)** Significance of correlations between ROS and RA parameters, the Spearman’s *P*-value is indicated for each panel.

Next, to test the impact of altered granulocyte ROS production on RA’s pathophysiology, spontaneous ROS production and the ROS indexes after STZ stimulation were tested for any correlations with DAS28, HAQ, CRP, and RF in new RA patients (Figures 3A,B). Both STZ peaks and STZ AUC correlated with DAS28 (*P* = 4 × 10^−5^ and *P* = 2 × 10^−7^, respectively), and CRP (*P* = 0.003 and *P* = 0.002, respectively). Regarding the time to reach the peak of ROS production, correlations were observed with CRP (*P* = 0.01). We conclude from these experiments that granulocyte ROS production upon STZ stimulation is altered in untreated eRA patients, and that a positive correlation exists between disease activity and STZ-dependent ROS production.

### Linkage of Certain Infections and ROS Parameters

Taking into account that ROS are major pathogenic molecules produced during viral infections, including V-URI and HSV, and at the same time, important players in the immune response against bacterial infections presented by tonsillitis in our groups, we next tested whether viral and/or bacterial infection symptom episodes might influence RA through the control of the NADPH oxidase (NOX) activity. Accordingly, we further evaluated the association between V-URI, HSV, and tonsillitis exacerbation episodes reported in the previous year with the basal level and STZ-dependent ROS production in new RA patients, relatives, and controls.

**Figure 4:**
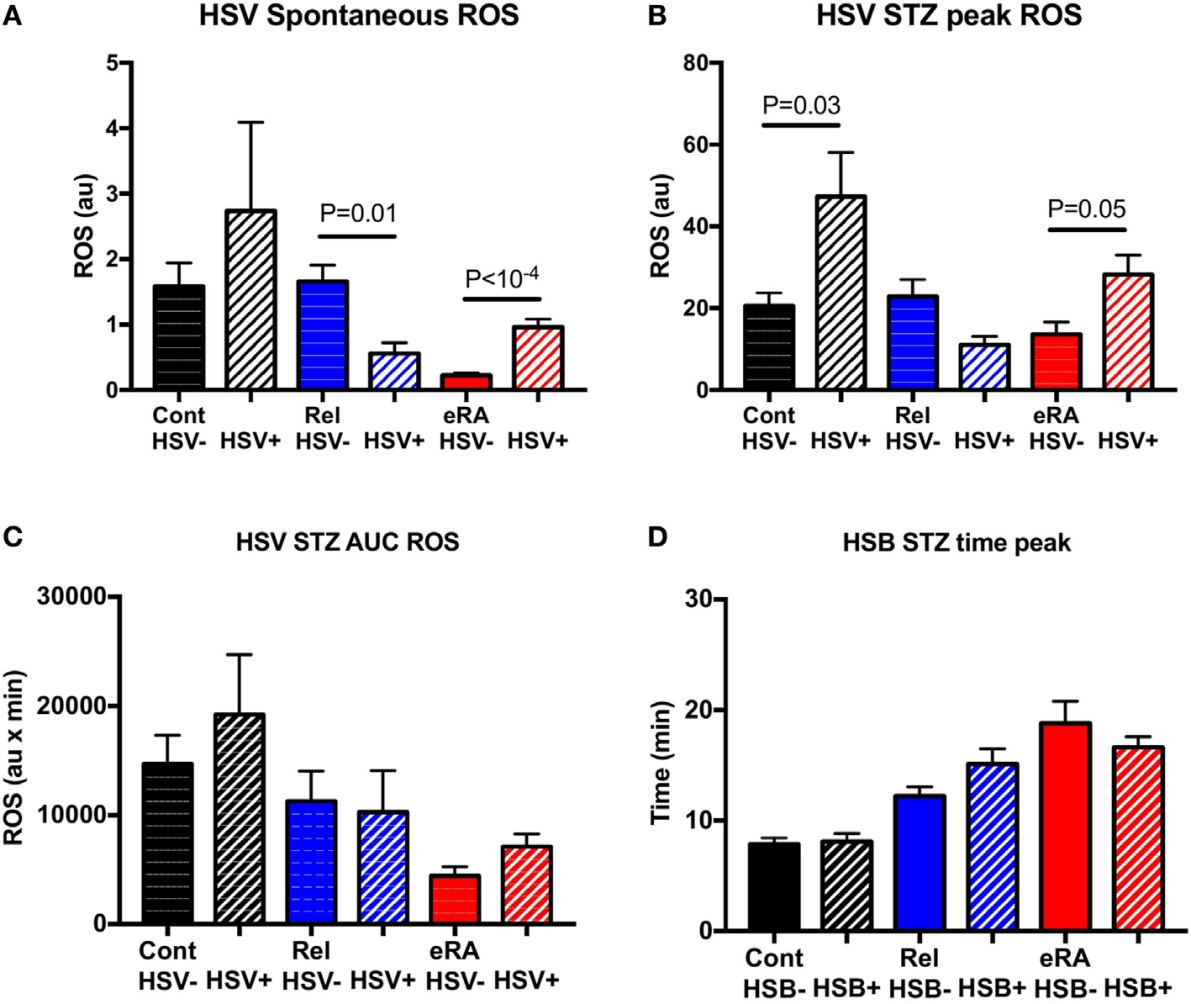
Impact of a history of herpes simplex virus (HSV) exacerbation/reactivation in the year preceding sample collection in granulocyte reactive oxygen species (ROS) production: spontaneous **(A)**, after serum-treated zymozan (STZ) stimulation at peak **(B)**, area under the curve (AUC) **(C)**, and time to reach peak ROS production **(D)**. Healthy controls (Cont), rheumatoid arthritis (RA) first-degree relatives (Rel), and untreated RA patients at onset (early RA). Statistics are indicated when *P* ≤ 0.05.

When eRA patients were dichotomized according to the appearance of HSV during the year before examination (Figure 4), the HSV exacerbation/reactivation episode history was associated with both increased spontaneous ROS production (*P* < 10^−4^) and, upon STZ stimulation, an increase in the peak of ROS production as observed in controls (*P* = 0.05 and *P* = 0.03, respectively). In first-degree relatives, an opposite association between an HSV episode history and spontaneous ROS production (*P* = 0.01) was highlighted, while no differences were reported following STZ stimulation.

**Figure 5:**
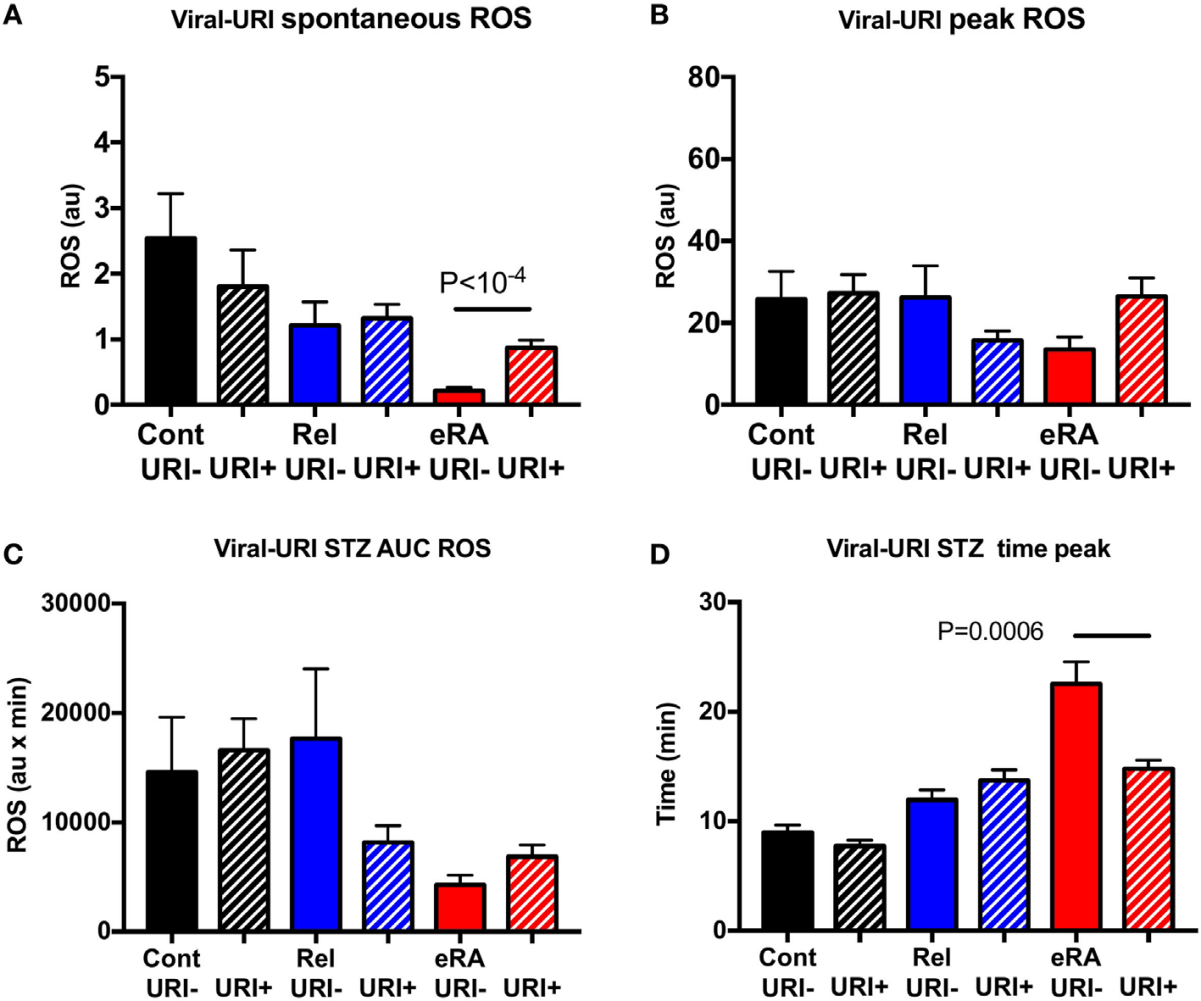
Impact of a history of viral upper respiratory tract infection symptoms (V-URI) in the year preceding sample collection in granulocyte reactive oxygen species (ROS) production: spontaneous **(A)**, after serum-treated zymozan (STZ) stimulation at peak **(B)**, area under the curve (AUC) **(C)**, and time to reach peak ROS production **(D)**. Healthy controls (Cont), rheumatoid arthritis (RA) first-degree relatives (Rel), and untreated RA patients at onset (early RA). Statistics are indicated when *P* ≤ 0.05.

V-URI episodes during the year preceding the study were associated in new RA patients with increased spontaneous ROS production (*P* < 10^−4^) and, upon STZ stimulation, to a normalization of the time to reach the peak (*P* = 0.0006) (Figure 5). No difference was observed for controls and relatives when considering the V-URI history.

**Figure 6:**
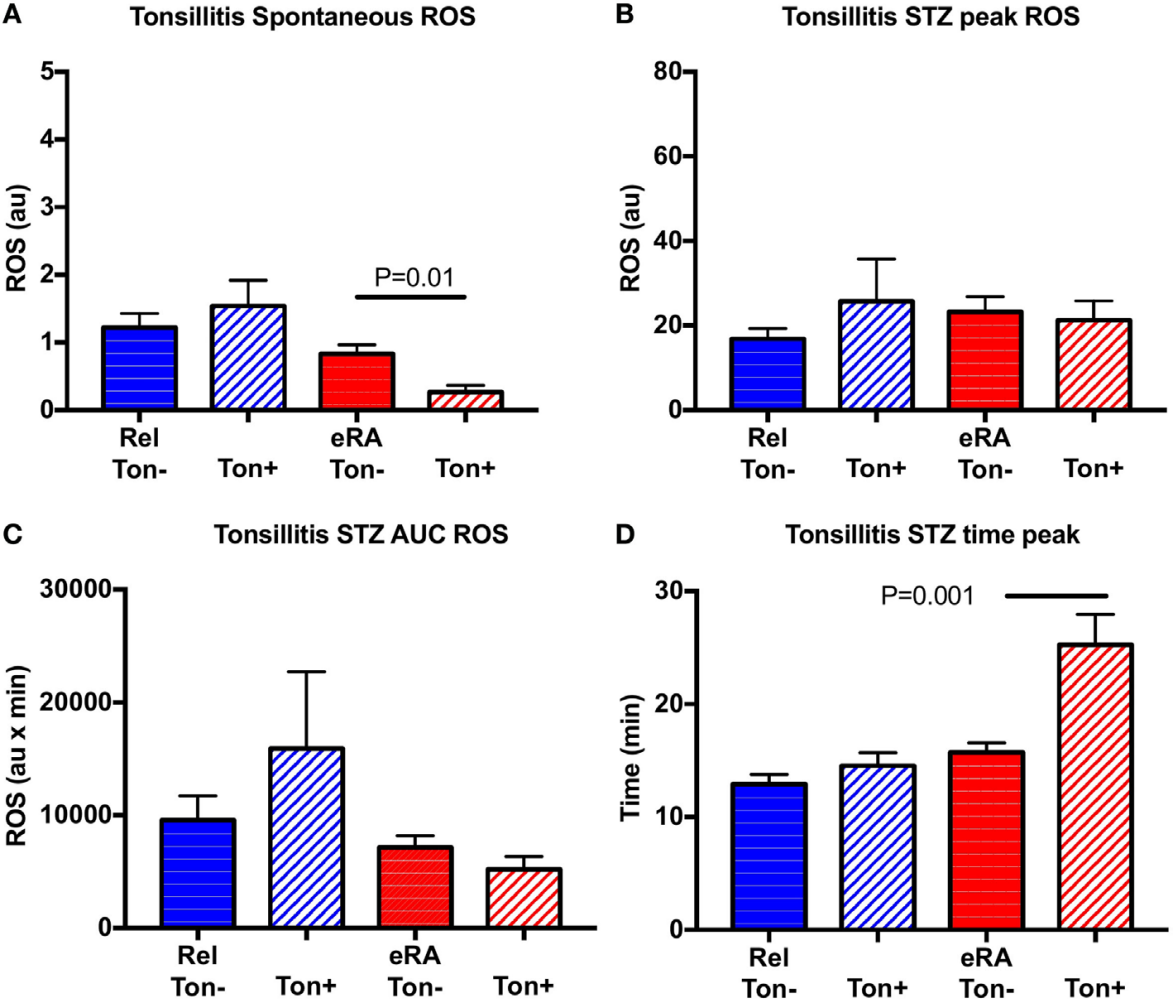
Impact of a history of chronic tonsillitis exacerbation in the year preceding sample collection in granulocyte reactive oxygen species (ROS) production: spontaneous **(A)**, after serum treated zymozan (STZ) stimulation at peak **(B)**, area under the curve (AUC) **(C)**, and time to reach peak ROS production **(D)**. Healthy controls (Cont), rheumatoid arthritis (RA) first-degree relatives (Rel), and untreated RA patients at onset (early RA). Statistics are indicated when *P* ≤ 0.05.

For chronic tonsillitis exacerbation, controls were not considered since only 2/58 controls presented episodes during the previous year. In contrast to HSV and V-URI, chronic tonsillitis exacerbation was associated in eRA patients with a negative impact on the spontaneous ROS production (*P* = 0.01) and an increase in the time to reach the peak (*P* = 0.001) (Figure 6). No differences were reported for the first-degree relative group.

## Discussion

Results from this study suggest an important contribution of the viral infectious episodes in the year preceding RA in women on both onset and activity of the disease after excluding patients with allergy and chronic viral diseases (viral hepatitis, HIV). First, and based on the incidence of the minor infectious episode history in patients with eRA, a critical role for HSV events may be suspected. Second, a history of HSV and V-URI episodes was associated with increased granulocyte ROS production and disease activity (HSV) or specific antibody production (V-URI) in untreated eRA patients, while a decrease was observed in the case of chronic tonsillitis exacerbation. Third, the link between ROS response and disease activity in eRA was further confirmed. Fourth, first-degree relatives of patients with eRA such as siblings, parents, and children presented an elevated incidence of viral infectious episodes with a normal immune response, characteristics that can help to better understand the mechanisms leading to RA.

### Infections and RA

Dangerous connections exist between infections and RA since, on the one hand, defective anti-infectious activity characterizes patients with RA while, on the other hand, infections are suspected of promoting autoimmunity (1). Among viral infections associated with autoimmunity, HSVs are strongly suspected of contributing to RA development, and we have observed an elevated rate of HSV events in the history of eRA patients. We have further established an association between HSV exacerbation/reactivation in the year preceding RA onset and disease activity (DAS28). These data are in agreement with previous reports. First, with regards to the patient’s personal and family history of RA, a higher personal history of HSV (OR = 2.4) is observed (12). Second, the incidence rate of HSV has already been demonstrated to be two times greater in RA than the rate observed in age matched controls (13). Third, a higher HSV viral load is also reported in RA patients (14), and this is interpreted as the result of impaired cellular immunity that characterizes RA patients. Fourth, the defective capacity of the immune system to control an HSV infection is considered to favor reactivation in patients with RA receiving biological or conventional disease-modifying antirheumatic disease drugs (15).

Similarly, among rhinovirus, enterovirus, respiratory syncytial virus, and influenza virus known to be associated with V-URI, the incidence of the latter is reported to be higher in RA than in controls and there is a 2.75-fold increase in pneumonia complications in RA patients infected with influenza (16). RA patients with interstitial pneumonia have elevated levels of anti-CCP and RF as recently reported (17). This is in agreement with our observation that a history of V-URI is associated with higher antibody prevalence (CCP2) in eRA patients supporting the concept that V-URI may be an actor in RA activity. However, we failed to confirm the higher prevalence of V-URI in eRA and such a discrepancy may be related to the seasonality of the infections and to the criteria for selection of the control cohorts between the studies.

The most studied bacterial strain associated with RA is related to *Porphyromonas gingivalis*, which is suspected of promoting autoimmunity by inducing protein citrullination, promoting HLA-DR overexpression and citrullinated peptide presentation to CD4 T cells, and by interfering with the production of cytokines and chemokines such as Jak/STAT and NF-kappaB (18, 19). In line with this model, case reports have been published indicating that RA disease can be improved following tonsillectomy (20), and that antibodies against a carbohydrate antigen, Strep A, of *Streptococcus pyogenes* are produced during chronic tonsillitis in RA patients (21). However (22), the analysis of 1,524 RA patients with associated antecedent tonsillectomy or appendectomy have failed to show any association suggesting that chronic tonsillitis is at the best an activator but not an inducer of RA, a conclusion that is in agreement with our observations showing that chronic tonsillitis exacerbations were increased in both new RA patients and their relatives.

### Infections and ROS

Host defense against microbial infection involves ROS production, and toll-like receptors (TLR) have been identified as a major class of pattern-recognition receptors necessary for triggering ROS. TLR family members expressed by granulocytes facilitate the recognition of pathogen-associated molecular patterns, as was demonstrated with bacterial pathogens that colonize tonsils (23). In the case of chronic inflammation and tissue injury, the host can in addition produce endogenous TLR ligands, known as damage-associated molecular patterns (DAMPs), and DAMPs have the potential to activate inflammation, initiating a vicious cycle in germ-free conditions (24). Abnormal expression of DAMPs is reported in human RA tissues, and it has been speculated that DAMPs are critical for RA pathogenesis by controlling granulocyte functions including the oxidative burst (25, 26). The DAMPs hypothesis is attractive since it provides an explanation to the major defective ROS activity observed in the basal level and after STZ activation (but not when using PMA for activation, data not shown) in RA patients. Another hypothesis is related to the fact that zymosan is a TLR2 ligand (27), and that earlier investigations have highlighted the importance of TLR2 function in RA pathogenesis (28, 29). Accordingly, the DAMP and/or TLR2 hypothesis can both explain the ROS dysregulation observed in eRA although cell samples were collected in periods without any clinical symptoms of an infection and without any routine laboratory signs of inflammation. Future experiments are necessary to test whether or not the abnormal STZ capacity to induce ROS in eRA patients is related to DAMPs and/or to an abnormal TLR2/NF-kB pathway.

Regarding viruses and, in particular, HSV, some of them alter granulocyte functions. Indeed, HSV can directly attach to granulocytes *via* the herpes virus entry mediator (HVEM) and formyl peptide receptors, penetrate into the cells, and subsequently increase ROS production (30). RA is characterized by HVEM overexpression on various cells due to the increased levels of phagocytosis, ROS production, as well as production of interleukin-8 (neutrophil chemoattractant) and TNF-alpha (31). ROS production might inhibit the antiviral immune response since, in particular, ROS downregulates NK cell function, NK cells being the principle players in the antiviral immune response and maintenance of the latent state of HSV (32, 33). The immune response during influenza infection, being an acute upper respiratory tract viral infection, includes such a strong granulocyte stimulation, and, in particular, the ROS production by these cells, that these cytotoxic factors become the most important drivers of the inflammatory process and its complications (34–37). In our study, we have observed an increased ROS activity when considering both spontaneous and STZ stimulated peak ROS activity of granulocytes from RA patients presenting a V-URI or HSV history in the preceding year. Since such an effect is also associated with higher RA activity (HSV) or a higher immune response (V-URI), three non-exclusive hypotheses can be proposed. First, there is a direct effect of the latent virus on granulocyte ROS production. Second, the effect is indirect and may be a consequence of the intensive inflammatory process, in this way, the HSV capacity to produce DAMPs has been reported (38). Third, since the pathogenic role of anti-Fc gamma receptor IIIb autoantibodies on granulocytes is well documented in RA, our data allow the assumption that there is some link between V-URI and RA associated autoantibodies (39–41).

While studying one of the essential mechanisms of anti-infectious activity, granulocyte ROS production in RA, a remarkable feature of NOX activity was revealed at eRA with a twofold decrease in the response to STZ stimulation. Granulocytes are known to be first responders to infections, and immediate ROS release in response to a pathogen is an important condition for curbing an infection. So, the NOX response rate against a pathogen might be essential for its successful immobilization. This is especially important during an aggressive bacterial infection. As a consequence, the slowing of the granulocyte response to STZ is suspected to play a role in the relatives’ and eRA patients’ susceptibility to the exacerbations of chronic tonsillitis, mainly caused by bacteria. At the same time, there was no difference in this index in the patients who underwent or did not undergo HSV event exacerbation since the rapid granulocyte ROS production does not play a significant role in the neutralization of viruses. It should be noted that previously we demonstrated the delayed monocyte NADPH oxidative response upon STZ stimulation as well (42).

### ROS and RA

In addition, we have observed an association between spontaneous ROS production, STZ stimulated ROS parameters and disease activity (DAS28, CRP) in patients with eRA at onset. However, the role of ROS in RA is not sufficiently clear. In the initial view, increased ROS activity has been documented in synovial joints to directly contribute to the inflammation, to induce the hyperplasia of synovial tissues, and to damage cartilage, bone, and ligaments (43). In addition, it was demonstrated that abnormal ROS activity controls antigen presentation and reduces T cell responsiveness in RA through effects on cell-surface proteins such as CD4 and signal transduction proteins such as LAT or Zap70 that are present close to the plasma membrane (44, 45). In addition, ROS, by damaging endothelial cells, increase the permeability of the vasculature and promote granulocyte migration to inflammation sites (90%). Last but not least, cytokine overproduction, including TNF-alpha and IL-1, is thought to be the main contributor to ROS in RA. However, the paradigm has changed with the discovery that allelic polymorphism in the respiratory burst oxidase component neutrophil cytosolic factor (ncf)1 in rat and ncf4 in human was associated with a more severe and a higher incidence of RA (46). The exact mechanisms are incompletely understood but may rely on an enhanced activation of autoreactive T and B cells as observed in ncf1 transgenic mice (47). In our study, a strong and positive correlation between disease activity (DAS28) and the level of ROS was observed, which is in agreement with a previous report that proposed measurement of ROS for monitoring disease severity in RA (48).

In conclusion and although patients with allergy and viral chronic infections were excluded from the study, we are aware of the limits of our study in terms of sample size, in terms of sex selection, and the fact that infections were established mostly on symptoms rather than biological parameters. Thus more studies including serological and an extended microbiota analysis are needed to support our observations and for that the longitudinal Tatarstan cohort study, which provides a longitudinal analysis of RA patients from the cohort of Tatarstan women and their first-degree relatives, is appropriate for the study of interactions between the multiple factors associated with RA.

## Ethics Statement

The study was approved by the Ethical Committee of the Kazan State Medical Academy, Kazan, Russia (Permit nr 1/2002).

## Author Contributions

All authors listed have made a substantial, direct, and intellectual contribution to the work and approved it for publication.

## Conflict of Interest Statement

The authors declare that the research was conducted in the absence of any commercial or financial relationships that could be construed as a potential conflict of interest. The reviewer AV and handling Editor declared their shared affiliation.
